# Temperature Profile in Starch during Irradiation. Indirect Effects in Starch by Radiation-Induced Heating

**DOI:** 10.3390/ma14113061

**Published:** 2021-06-03

**Authors:** Mirela Braşoveanu, Monica R. Nemţanu

**Affiliations:** Electron Accelerators Laboratory, National Institute for Lasers, Plasma and Radiation Physics 409 Atomiştilor St., P.O. Box MG-36, 077125 Bucharest-Măgurele, Romania; mirela.brasoveanu@inflpr.ro

**Keywords:** electron beam, corn starch, potato starch, moisture content, specific heat capacity, pH, color parameters

## Abstract

Present research deals with exposure of granular starch to the accelerated electron of 5.5 MeV energy in order to examine: (i) the temperature evolution in starch within an irradiation process and (ii) the indirect effects generated in starch by radiation-induced heating. The temperature evolution in potato and corn starches within the irradiation process was investigated by placing two different sensors inside each starch batch and recording the temperature simultaneously. Each starch batch was sampled into distinct location sectors of different absorbed radiation levels. The output effects in each sample were analyzed through physicochemical properties such as moisture content, acidity and color attributes. The outcomes showed that a starch temperature profile had different major stages: (i) heating during irradiation, (ii) post-irradiation heating, up to the maximum temperature is reached, and (iii) cooling to the room temperature. A material constant with signification of a relaxation time was identified by modeling the temperature evolution. Changes of the investigated properties were induced both by irradiation and radiation-induced heating, depending on the starch type and the batch sectors. Changes in the irradiated batch sectors were explained by irradiation and radiation-induced heating whereas changes in the sector of non-irradiated starch were attributed only to the heating.

## 1. Introduction

Starch is one of the most widespread natural polymers, being composed of two different fractions: amylose (linear fraction) and amylopectin (branched fraction). Starch is used in various food and non-food applications such as pharmaceuticals and biomedical products, packaging materials, textiles, adhesives, etc. Native starch has limited functionality in technological applications due to its poor processing properties such as high viscosity, tendency for retrogradation, lack of thermal stability. However, it has a range of very significant advantages being a cheap, renewable, non-toxic, and widely available biodegradable raw material [1]. Thus, the need for starch modified by using emerging ecological techniques has gradually increased worldwide in recent years [2]. One of these techniques is based on ionizing radiation (gamma rays or electron beams) through which starch can be easily modified by degradation, crosslinking or grafting processes [3,4,5,6].

Electron beam irradiation is widely used in material processing, involving both chemical and thermal effects. The chemical effects due to ion and free radical generation [7,8] are connected with radiation chemistry. The thermal effects are especially related to the metal and alloy refining, melting and welding applications [9,10,11], where the transformation of the radiation energy into heat is done deliberately. It is to be remembered that the overlapping of thermal effects and radiation-induced chemical effects is avoided most of the times, especially for heat-sensitive materials. Among heat-sensitive materials can be found biological materials, which are sensitive to temperature variation, as well as materials that undergo phase transitions in temperature ranges slightly higher than the ambient temperature. As an example, granular starch undergoes an irreversible transition known as gelatinization in the presence of sufficient moisture above a certain temperature, generally between 60 and 80 °C [12]. In this context and in the absence of knowledge on this topic in literature, there is reason to believe that the heat transferred to starch from radiation field can make additional contribution to changes of the starch properties by irradiation. The water content of the starch can be diminished by evaporation due to the developed heat during irradiation. In this way, the reaction environment would be changed as well as the specific heat capacity of the starch. All these aspects actually underline the importance of knowing the dynamics of the temperature in starch during irradiation in order to control the final results of irradiation. In our previous work [13] on this topic, we reported a first theoretical approach concerning the temperature distribution in the granular corn starch exposed to electron beam irradiation through a semi-analytical model based on the heat equation in Cattaneo-Vernote formalism and solved by using the integral transform technique on finite domains. To the best of our knowledge, there are no other studies that explore this thematic area.

Furthermore, in the present work, we aimed out to show the evolution of temperature in granular starch subjected to an irradiation process. At the same time, the presence of indirect effects produced in starch by electron-beam induced heating was highlighted. For this purpose, starches from two different botanical sources, potato and corn, were exposed to an accelerated electron field in a selected irradiation setup (geometry and irradiation parameters). For both types of starch, the temperature was measured simultaneously at two points in the batch. The analysis of batches was performed by sampling them into three distinct location sectors and evaluating some physicochemical properties such as moisture content, pH and color attributes. The findings of this work are relevant for any research related to the irradiation of starch-based materials, especially whenever the research results are transferred at a large scale. An important advantage in the radiation application comes from the possibility of irradiating samples with large dimensions, but in this case the cooling process is more difficult and unforeseen thermal effects can occur.

## 2. Materials and Methods

### 2.1. Materials

Potato starch (S4251; moisture content: ~18%) and corn starch (S4126; moisture content: ~11%) used in the experiments were purchased from Sigma-Aldrich Company (St. Louis, MO, USA).

### 2.2. Electron Beam (E-Beam) Irradiation

The irradiation of starch powder in solid state was performed in static mode by using an e-beam generated by a linear accelerator ALID-7 of energy of 5.5 MeV (NILPRP, Bucharest-Măgurele, Romania). The electron accelerator facility is of traveling-wave type which uses microwaves in the S-band at 2.99 GHz propagating in a disk-loaded tube of about 2 m long, and the microwaves are produced by an EEV-M5125 type magnetron delivering 2 MW of power in pulses of 4 μs [14,15]. The ALID-7 accelerator is used for various applied radiation researches [16,17,18]. The e-beam had a Gaussian-like profile of dose rate distribution, which was canceled at a distance of *r* = 37 mm from the beam axis at a distance of 220 mm from accelerator window exit. The maximum dose rate of 𝒟*_o_* = 12 kGy/min in the beam center, on the irradiation sample surface, was reached by using a mean beam intensity of 4 µA. The sample irradiation was carried out for a period of 435 s, at the room temperature (22 ± 1 °C) and ambient pressure in air.

### 2.3. Irradiation Geometry and Temperature Measurement

**Figure 1:**
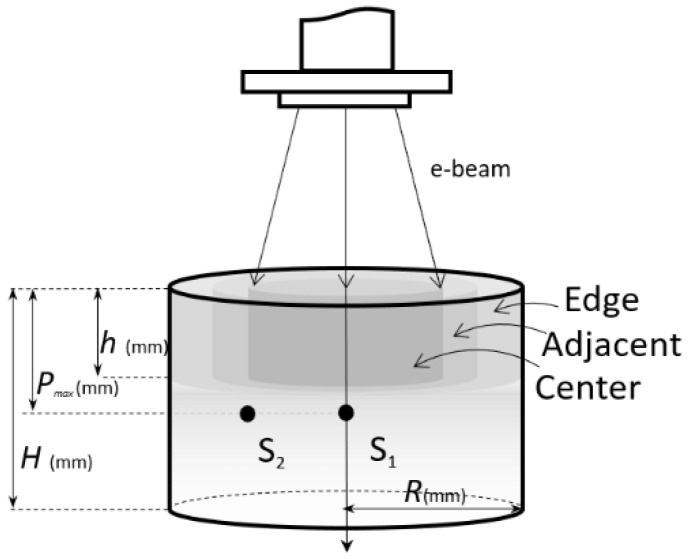
Experimental set-up.

The starch powder was placed in a cylindrical cardboard box (Figure 1) so that the density was 540 ± 10 kg/m^3^ for both types of starch. Consequently, the maximum depth of electrons in starch determined according to the ISO/ASTM 51649:2002(E) [19] was *P_max_* = 55 mm. The box had the radius *R* = 65 mm and the height *H* = 100 mm and its symmetry axis coincided with the e-beam axis during irradiation. Two temperature sensors (K-type thermocouple of a multifunctional digital multimeter DVM891, Velleman^®^, Gavere, Belgium) were placed inside the starch batch: S1 on the e-beam axis and S2 at *r* = 37 mm from the axis. Both sensors were located at a depth of 55 mm, which is equal to the maximum depth of the electrons in starch, in order to avoid that these sensors directly absorb heat/energy from the radiation field.

The temperature values were simultaneously recorded by both sensors during starch irradiation and after irradiation, namely during the heating due to the radiation field as well as during the cooling toward room temperature.

### 2.4. Sampling of Batches

Each batch of irradiated starch was divided into three distinct sectors, concentric around the symmetry axis. Each starch sample was collected from each location sector to a depth of *h* = 40 mm (Figure 1). The collected samples were then investigated separately.

The sector named Center, located 37 mm around the center and exposed to the proper absorbed dose rate and the highest heating, was associated with the temperature recorded by the sensor S1. Thus, S1_potato_ and S1_corn_ refer to the temperature in this sensor for potato starch sample and corn starch sample, respectively.

The second sector, named Adjacent, was located around the Center, up to 50 mm from the beam symmetry axis, being associated with the temperature recorded by the sensor S2. Thus, S2_potato_ and S2_orn_ refer to the temperature in this sensor for potato starch sample and corn starch sample, respectively. A “residual” absorbed dose rate in the Adjacent sector was estimated to be less than 7% from the central maximum dose rate due to the secondary electrons.

The Edge sector was located between 50 and 65 mm from the center, at the periphery of the sample, free of radiation, being associated with a small variation of temperature, but not recorded.

### 2.5. Moisture Content Determination

The moisture content of the samples was measured by using an IR-200 moisture analyzer (Denver Instruments Company, Denver, CO, USA) at 105 °C for 45 min.

### 2.6. pH Measurement

Starch samples were mixed in distilled water by magnetic stirring on bath water at 85 °C for 30 min, and the mixtures of 1% (*w/v*) concentration were then allowed to stand at room temperature (25 ± 1 °C). After cooling, pH was measured at 25 ± 1 °C with an inoLab^®^ 7110 pH-meter (WTW, Weilheim, Germany).

### 2.7. Optical Measurements

Starch samples were mixed in distilled water by continuously magnetic stirring on bath water at 85 °C for 30 min, and the mixtures of 1% (*w/v*) concentration were then allowed to stand at room temperature (25 ± 1 °C). After cooling, the UV-Vis spectra of the samples were recorded in a Cary 100 Bio spectrophotometer (Varian, Inc., Walnut Creek, CA, USA). Colorimetric features of the samples were measured in absorbance in the visible region (360–830 nm), for standard illuminant D65 (daylight source), observer angle of 10° (perception angle of a human observer). The colorimetric attributes expressed as CIELCH parameters (L*, C*, h°) were analyzed by using the Color Application of Cary Win UV v. 3.10 software (Vary, Inc., Walnut Creek, CA, USA, 2006).

### 2.8. Statistical Approach

The results reported are expressed by means of values ± standard deviation of triplicate determinations. The processing of experimental data was performed using OriginPro 8.1 (OriginLab Corporation, Northampton, MA, USA, 2016), Microsoft^®^ Excel 2010 (Microsoft Corporation, Redmond, WA, USA), and InfoStat versión 2018 [20]. The data were analyzed by using analysis of variance with Fisher LSD (least significant differences) post-hoc test to discern the statistical difference. A probability value *p* ≤ 0.05 was considered as statistically significant.

## 3. Results and Discussion

### 3.1. Temperature Profile

**Figure 2:**
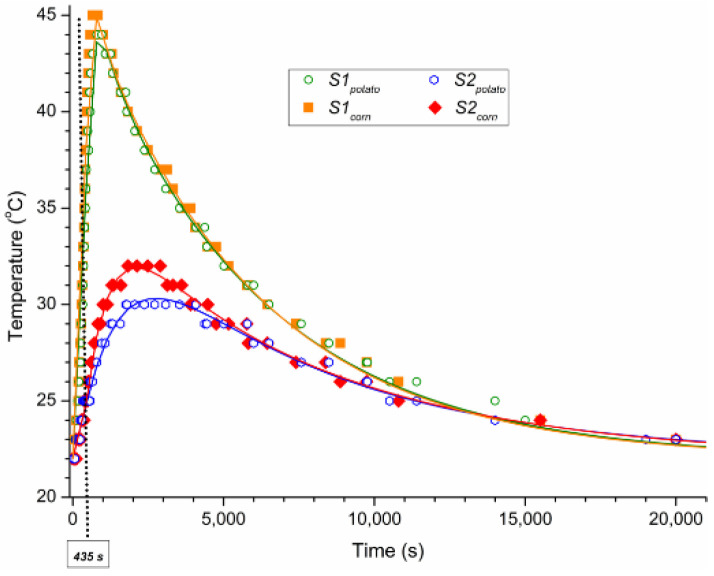
Temperature profiles through sensors S1 and S2 for both irradiated potato and corn starches.

The evolution of the temperature recorded by the two sensors, for each type of starch, during irradiation and cooling is presented in Figure 2. It was observed that there are three major stages: (i) heating during irradiation, up to *t* = 435 s, (ii) post-irradiation heating, up to the maximum temperature is reached, and (iii) cooling to the room temperature. This finding is consistent with the fact that the heat continues to propagate in starch for a while, in connection with the place where the sensors are placed, after the irradiation process is stopped and before the cooling process begins [13]. The maximum temperature and the moment of its reaching differ for the two types of investigated starch, both in the first sensor, S1_potato_ and S1_corn_, but even more visible in the second sensor, S2_potato_ and S2_corn_. This differentiation of behavior can be attributed to the different thermal properties of these two types of investigated starch.

During the irradiation stage, the potato starch had a lower heating rate than corn starch, reaching a temperature of 36 °C in S1_potato_ compared to 38 °C in S1_corn_ after 435 s. Also, the potato starch continued to heat up post-irradiation for a longer time than corn starch, visible in both sensors. Therefore, the potato starch needed 364 s to reach the maximum temperature in S1 while the corn starch needed only 216 s. Conversely, the maximum temperature was higher for corn starch compared to the potato starch, with 1 °C in S1, and with 2 °C in S2. However, the time to reach the peak temperature in S2 appeared to be comparable for the two starches, but this maximum was maintained much longer for potato starch than corn starch. In this way, the last stage started later with lower values for potato starch than corn starch, in both sensors. The cooling curves showed an exponential trend, so that the temperatures for potato starch and corn starch in the first sensor, S1_potato_ and S1_corn_, respectively, overlapped quite well, especially after about 2000 s. Also, in the second sensor, the cooling process was similar for the two starches after about 5000 s.

By analyzing the cooling curves, it can be noticed that their evolution followed an exponential law, in each sensor:

(1) $$T=T_{0}e^{-t/\tau_{0}}+22,$$

where *T* is the temperature in each sensor during cooling process (°C), *T*_0_ is a temperature parameter (°C), which depends on both the maximum temperature and the time considered as the beginning of the cooling process, *t* is the time (s), *τ*_0_ is a parameter that has the signification of a relaxation time (s), namely the time required for starch to decrease the temperature to the equilibrium temperature e times.

The values of *τ*_0_ obtained by fitting the experimental data of the cooling process are displayed for each starch and each sensor in the first row of Table 1. The parameter τ_0_ estimated in this way can characterize the starch thermal behavior, but also it depends on the geometry characterizing the starch at the time of temperature measuring.

On the other hand, we were able to find a function that also describes the evolution of the starch temperature for post-irradiation heating process:

(2) $$T=\left[T_{i}\;\frac{t-\Delta t}{\tau_{i}}\;e^{-t/\tau_{i}}+T_{0}\right]e^{-t/\tau_{0}}+22,$$

where *T* is the temperature in each sensor after irradiation stopping (°C), *T_i_* (°C), *τ_i_* (s) and Δ*t* (s) are parameters that characterize the post-irradiation heating, *t* is the time (s), *T*_0_ is a temperature parameter, associated with the temperature at which the cooling started, *τ*_0_ is the parameter with significance of relaxation time (s).

Keeping the values of *τ*_0_ found for the cooling process, we determined the other parameters that best fit (*R*^2^) the experimental data for the post-irradiation heating processes, and the values obtained are shown in Table 1. By carefully evaluating the determined parameter values, we noticed that *T*_0_ is the difference from the room temperature to the maximum value reached in each sensor, and *τ_i_* can express the time required to reach the maximum temperature after irradiation in each sensor. *T_i_* and Δ*t* take values that describe the temperatures in sensors when the irradiation is off as well as the shape of the curves around the maximum value. The parameters *T_i_, τ_i_*, and Δ*t* depend more on the irradiation geometry (S1 vs. S2) than on the type of starch (S1_potato_ vs. S1_corn_ and S2_potato_ vs. S2_corn_, respectively). Note that even though *τ*_0_ and *τ_i_* depend on the starch type and geometry, the values of their difference *τ*_0_ − *τ_i_* are not significantly different. This finding suggests that *τ*_0_ − *τ_i_* could be a material constant, and the starch relaxation time could actually have values around 5800 s.

### 3.2. Moisture Content and Specific Heat Capacity

The moisture content for the starch sampled from the three considered locations (Center, Adjacent and Edge) was evaluated for each type of starch (potato and corn) together with the corresponding control samples. The results presented in Table 2 show that the moisture content of both types of starches is insignificantly (*p* > 0.05) affected in the Edge compared to that of the control samples. A decrease in moisture content was observed in the Center and Adjacent sectors compared to the control samples for both starches. However, corn starch showed a drastic decrease (*p* ≤ 0.05) of moisture content only in the Center. At the same time, the potato starch, with a higher moisture content, suffered (*p* ≤ 0.05) the highest loss, of over 20% in the *Center*. It is noteworthy that potato starch showed a higher moisture loss than corn starch for each sector, which can be explained by the origin of different botanical sources and, implicitly, their different structural arrangement.

The starch dehydration could be explained by a water vapor transport that accompanied the cooling process which occurred by heat dissipation from the Center to the Edge. Moisture content losses in the Center and Adjacent sectors are in accordance with the estimated values for the temperature parameter *T*_0_. Thus, these values are associated with the maximum temperature reached in the sensors assigned to these location sectors. The different temperatures measured in a sensor for investigated samples could be explained by their different specific heat capacities, *c_p_*. It is well known that the specific heat capacity *c_p_* of a sample is directly related to its moisture content. Considering that *c_p_* = 3.35 *u* + 0.84 (kJ kg^−1^ K^−1^) [21], where *u* = *u%*/100 is the mass ratio of water in starch (kg kg^–1^), the specific heat capacity values *c_p_* were determined as shown in Table 2. Since moisture content was diminished due to irradiation, the specific heat capacity and, consequently, the thermal behavior of the samples were affected during irradiation and post-irradiation.

### 3.3. pH Evaluation

The native starches had an acidic character with a pH around 5.5 for both starches. Both starches showed the reduction (*p ≤* 0.05) of pH values for all investigated location sectors in comparison to the control sample (Table 3). Although both types of starches had a similar pH value decreasing in the Center, the corn starch suffered a greater reduction in pH value compared to potato starch for the Adjacent. The decreasing pH by e-beam irradiation in the presence of oxygen could be assigned to the fragmentation of starch molecules due to the free radicals and oxidative reactions leading to the formation of compounds with acidic chemical groups [22,23].

On the other hand, only potato starch had a significant reduction in pH value in the Edge in comparison to control sample. However, it should be noted here that the Edge sector was considered free of radiation, but with a small temperature variation, which led us to believe that the difference (*p ≤* 0.05) in pH value for potato starch can be totally attributed to a thermal phenomenon, even if it was a small one. This different behavior of the two types of starch can be attributed to their different amylose content. It is known that the thermal properties of starches from different botanical sources are influenced not only by the structural features, but the amylose content or amylopectin/amylose ratio, which leads to higher or less thermal stability of starch polymers [24,25]. There are reports showing that thermal stability decreased with increasing amylose content for both corn starch [26] and potato starch [27]. As the potato starch has a lower amylose content than the corn starch [28,29] and therefore it may show a greater thermal sensitivity, the change (*p ≤* 0.05) in its pH value due to the heating in the Edge can be explained.

### 3.4. Color Parameters

In the CIE L*C*h° coordinate system, L* is lightness (0 (black) → 100 (white)), chroma (saturation) C* is the quantitative component of the color while hue *h°* is the qualitative component of color, being expressed in degrees: 0° (red), 90° (yellow), 180° (green), 270° (blue). In these terms, native potato starch showed higher values of L* and C* than native corn starch. In other words, the potato starch had greater transparency and saturation in comparison to corn starch that showed the tendency of its color to grey. Instead, the hue *h°* had similar values indicating the same slightly yellowish color hue for both native starches.

In our experiment, it was observed that the samples from the considered location sectors, for both starches, showed modified values *(p* ≤ 0.05) for the parameters that characterize the color space L*C*h° compared to the corresponding native counterparts. However, the corn starch from Edge showed values of color parameters similar to those of its native counterpart.

**Figure 3:**
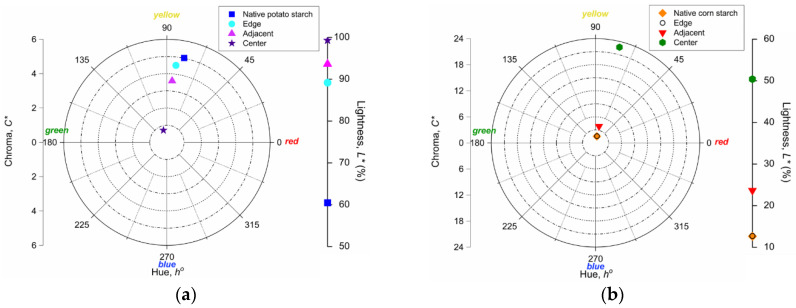
CIE L*C*h° parameters of the investigated samples: (**a**) potato starch and (**b**) corn starch.

Although the color parameter evolution in opposite directions was noted for both potato and corn starches in the Adjacent and Center, all samples of both starches showed an intensification in terms of lightness by irradiation (Figure 3). Similar observations were previously reported by Nemţanu & Braşoveanu [30].

In the case of potato starch (Figure 3a), the chroma C* decreased significantly while the hue *h°* tended to green by moving from the red-yellow quadrant (0–90°) to the yellow-green one (90–180°) for the sample from the Center. In contrast, for corn starch (Figure 3b), the color saturation C* of the sample from the Center intensified dramatically, and the hue *h°* practically had only a slight shift to red (~2.5° for the sample from the Center), indicating the red-yellowish color trend.

In this study of the chromatic attributes, as in the case of pH, it was noticed that the potato starch from Edge showed significant changes compared to its native counterpart. Therefore, once again, it was found that potato starch, unlike corn starch, was affected by a thermal phenomenon induced by irradiation in a location sector (Edge) considered free of radiation.

Based on all these results and observations, it can be stated that radiation-induced heating can affect the physicochemical properties of starches, depending on their botanical source, moisture content, amylopectin/amylose content, and thermal sensitivity.

## 4. Conclusions

The temperature evolution in two different granular starches (potato and corn) within an electron beam irradiation process was investigated. Also, the indirect effects that occurred in starch by radiation-induced heating were studied by sampling each starch batch into distinct location sectors having different absorbed radiation levels (maximum dose rate, “residual” dose rate, no radiation). The main findings of this study are:

1. The temperature profiles recorded in starch samples showed three major stages: (i) heating during irradiation, (ii) post-irradiation heating, up to the maximum temperature is reached, and (iii) cooling to the room temperature.
2. The maximum values of temperatures reached and the heating rates were different for both starches in the studied location sectors. However, the cooling rates were similar for both starches in each location sector thermally studied.
3. The evolution of the measured temperatures was modeled as a function of the parameters that depend on the starch type and the location sector considered inside the batch. Based on this modeling, a material constant having the significance of a relaxation time was identified with values around 5800 s.
4. Dehydration and changes in the values of the specific heat capacity, pH and color parameters of the starch were noticed due to the irradiation and radiation-induced heating, depending on the starch type and the batch sectors. The changes in the irradiated batch sectors (with maximum or “residual” dose rate) could be explained by irradiation and radiation-induced heating. On the other hand, the changes in the sector where the starch was practically not irradiated could be attributed only to the heating. Although the reached temperatures here were lower than in the other batch sectors, these changes cannot be ignored.

Therefore, for a heat-sensitive material like starch, specific experimental precautions are required to prevent the temperature rise and dehydration due to irradiation whenever indirect consequences are not desired. Moisture-containing materials may undergo a dehydration process by irradiation as a result of the transport of water vapors. At the same time, some thermal characteristics such as specific heat capacity can modify considerably due to the very high value of the water heat capacity. Our findings are of practical use for any experimental design in which the radiation-induced direct and indirect effects in the moisture-containing materials should be taken into consideration. When simulating processes due to irradiation, such as Monte Carlo method, it is necessary to take into account such variations in temperature and moisture content that can occur in a material. Thus, the main challenge in this research field is the enhanced control and mitigation of the radiation-induced thermal effects in order to enable the desired chemical effects on starch-based materials. Future work in the field should be performed to differentiate the effects induced in starch directly by irradiation from those developed indirectly by radiation-induced heating.

## Figures and Tables

**Table 1:** Values of the parameters in the Equations (1) and (2) as result of the experimental data fitting.

Parameter	Potato Starch		Corn Starch	
	S1_potato_	S2_potato_	S1_corn_	S2 _corn_
*τ*_0_ (s)	6260 ± 108	7585 ± 409	5981 ± 116	6964 ± 248
*τ_i_* (s)	381 ± 31	1914 ± 448	267 ± 22	1117 ± 252
*τ*_0_ − *τ_i_* (s)	5879 ± 139	5671 ± 857	5714 ± 138	5847 ± 500
*Δt* (s)	670 ± 19	3993 ± 1675	627 ± 15	3834 ± 2126
*T*_0_ (°C)	23.0 ± 0.3	13.1 ± 0.7	24.6 ± 0.2	14.7 ± 0.3
*T_i_* (°C)	40.9 ± 3.6	6.9 ± 4.0	45.5 ± 4.9	5.7 ± 4.1
*R* ^2^	0.9944	0.9754	0.9973	0.9866

**Table 2:** Values of the moisture content (*u*%) and specific heat capacity (*c_p_*) for studied starches.

Location Sector	Potato Starch		Corn Starch	
	*u* (%)	*c_p_* (J kg^−1^ K^−1^)	*u* (%)	*c_p_* (J g^−1^ K^−1^)
Control sample (native)	17.7 ± 0.5 ^a^	1434 ±15 ^a^	11.2 ± 0.3 ^a^	1215 ± 10 ^a^
Edge	17.5 ± 0.1 ^ab^	1429 ± 5 ^ab^	11.2 ± 0.4 ^a^	1216 ± 12 ^a^
Adjacent	16.5 ± 0.4 ^b^	1395 ± 14 ^b^	10.6 ± 0.4 ^a^	1196 ± 12 ^a^
Center	13.9 ± 0.4 ^c^	1308 ± 14 ^c^	9.1 ± 0.5 ^b^	1147 ± 17 ^b^

Values within each column with different superscripts are significantly different (*p ≤* 0.05).

**Table 3:** pH values for investigated starch samples.

Location Sector	Potato Starch	Corn Starch
Control sample (native)	5.56 ± 0.06 ^a^	5.43 ± 0.09 ^a^
Edge	5.14 ± 0.18 ^b^	5.32 ± 0.12 ^a^
Adjacent	4.41 ± 0.19 ^c^	4.12 ± 0.05 ^b^
Center	3.57 ± 0.04 ^d^	3.54 ± 0.03 ^c^

Values within each column with different superscripts are significantly different (*p ≤* 0.05).

## Data Availability

Data sharing is not applicable for this article.
